# Prize Essay

**Published:** 1855-01

**Authors:** 


### PRIZE ESSAY.

In the September number of the *New York Medical Times*, a report is given of a clinical lecture in the New York Hospital, in which is described two cases of ununited fracture, treated after what the Surgeon (Dr. Markoe) called the method of Dr. Brainard, of Chicago. One of the cases it appears was successful—but Dr. Detmold, a Surgeon of some distinction in that city, claims, and as his letter below would seem to show, very justly, a priority in the idea and practice. The operation consists in making several perforations through the ends of the fragments of bone by a common drill through a single opening in the shin. Dr. Detmold's note to the Editors of the *Medical Times* on the subject published in the October number is as follows:

"New York, Sept. 4th, 1854.

GENTLEMEN:—In the last number of the *Times*, Dr. Markoe in his clinical remarks, speaks of an operation for ununited fracture, and says, "I propose to adopt a plan which was first suggested to us by Dr. Brainard, of Chicago," etc. Now I have no fault to find with the *first* suggestion, but the operation I claim as original and as mine. I performed it successfully four years ago upon a patient who was dismissed from the City Hospital with ununited fracture of the tibia, and presented the case before the New York Academy of Medicine.* In another case, likewise dismissed from the City Hospital, I requested the New York Academy of Medicine to appoint a committee to witness the operation and its results, which was done and reported upon. The first case was referred to, if I mistake not, in the very first number of the *Times*, by one of the Surgeons of the City Hospital, and a slur thrown upon the operation or the operator, I forget which. I have lately conversed with Dr. Brainard on the subject, and convinced him of the priority of my operation.

Very respectfully yours, &c.,

WM. DETMOLD, M. D."

Some time since Dr. Brainard reported as novel, the treatment of the bite of the Rattle Snake with Iodine. A practitioner at Joli-

*This case and the operation were reported in several Medical Journals.

et in Ill., soon after showed that he had reported cases treated successfully by Iodine some time before—and since then it has been proved that another physician in Ill. preceded them both in the published use of the remedy; and we should not be at all surprised if when the noise of this controversy spreads abroad, some other Surgeon comes forward and proves that neither of the contestants is entitled to the claim of originality.

We have indeed, a vague impression that we heard something of a similar operation some years ago, (perhaps it was Dr. Detmold's publication) and shall wait with a degree of curiosity the results of the discussion. The fact that a prize was awarded the essay describing this method, by the National Medical Association in May last, gives the subject additional interest.—*Peninsular Journal of Medicine.*

